# Seabird-affected taluses are denitrification hotspots and potential N_2_O emitters in the High Arctic

**DOI:** 10.1038/s41598-018-35669-w

**Published:** 2018-11-22

**Authors:** Kentaro Hayashi, Yukiko Tanabe, Keisuke Ono, Maarten J. J. E. Loonen, Maki Asano, Hirotsugu Fujitani, Takeshi Tokida, Masaki Uchida, Masahito Hayatsu

**Affiliations:** 1 Institute for Agro-Environmental Sciences, National Agriculture and Food Research Organization, 305-8604 Tsukuba, Japan; 2 National Institute of Polar Research, 190-8518 Tachikawa, Japan; 3 SOKENDAI (The Graduate University for Advanced Studies), 190-8518 Tachikawa, Japan; 4 Arctic Centre, University of Groningen, 9700 AS Groningen, The Netherlands; 5 Faculty of Life and Environmental Sciences, University of Tsukuba, 305-8572 Tsukuba, Japan; 6 Research Organization for Nano and Life Innovation, Waseda University, 162-8480 Tokyo, Japan

## Abstract

In High Arctic tundra ecosystems, seabird colonies create nitrogen cycling hotspots because of bird-derived labile organic matter. However, knowledge about the nitrogen cycle in such ornithocoprophilous tundra is limited. Here, we determined denitrification potentials and *in-situ* nitrous oxide (N_2_O) emissions of surface soils on plant-covered taluses under piscivorous seabird cliffs at two sites (BL and ST) near Ny-Ålesund, Svalbard, in the European High Arctic. Talus soils at both locations had very high denitrification potentials at 10 °C (2.62–4.88 mg N kg^−1^ dry soil h^−1^), near the mean daily maximum air temperature in July in Ny-Ålesund, with positive temperature responses at 20 °C (Q10 values, 1.6–2.3). The talus soils contained abundant denitrification genes, suggesting that they are denitrification hotspots. However, high *in-situ* N_2_O emissions, indicating the presence of both active aerobic nitrification and anaerobic denitrification, were observed only at BL (max. 16.6 µg N m^−2^ h^−1^). Rapid nitrogen turnover at BL was supported by lower carbon-to-nitrogen ratios, higher nitrate content, and higher δ^15^N values in the soils at BL compared with those at ST. These are attributed to the 30-fold larger seabird density at BL than at ST, providing the larger organic matter input.

## Introduction

Arctic tundra ecosystems are experiencing rapid warming due to climate change^1–4^. The soil in tundra ecosystems contains large stocks of organic matter, carbon (C), and nitrogen (N) because the cold and wet climate limits organic matter turnover^1^. Tundra ecosystems are therefore under limitation of inorganic N such as ammonium and nitrate, like many other terrestrial ecosystems^5–8^. However, mineralisation in mesic meadow tundra produces a large amount of ammonium^9^, suggesting that N turnover in tundra ecosystems can vary depending on the local conditions. The rapid warming due to climate change most likely further perturbs organic matter and N turnover in Arctic tundra ecosystems.

Nitrogen turnover is controlled by various processes comprising N cycle. For example, organic N is decomposed to ammonium by mineralisation, part of which is then oxidised to nitrate, via nitrite, by nitrification; both are regulated by microbial processes^10–13^. Ammonium and nitrate are also input from the atmosphere to terrestrial ecosystems by deposition^14,15^. Denitrification is the series of microbial processes that reduce nitrate to dinitrogen (N_2_) via nitrous oxide (N_2_O) in an anaerobic condition; these processes require nitrate and organic matter as substrates^10–13^. Denitrification returns N from the terrestrial ecosystem to the atmosphere through the emission of N_2_ and N_2_O.

N_2_O is an environmentally important compound because of its strong positive radiative forcing and ability to deplete stratospheric ozone^16,17^. In anaerobic soils, N_2_O is produced mainly by denitrification^10,12,13^, as well as by nitrification in aerobic soils and other processes^11^. Nitrification provides an important supply of nitrate as the substrate for denitrification^18,19^. N_2_O in soil is reduced to N_2_ under a strong anaerobic condition (complete denitrification). Under a partial anaerobic condition, N_2_O production outweighs the consumption and a part of the excess N_2_O in soil is released to the atmosphere^11,20^. Soils under natural vegetation are the world’s largest source of atmospheric N_2_O, accounting for 37% of global emissions (6.6–17.9 Tg N yr^−1^)^16^.

Although tundra soils are traditionally considered as poor N_2_O emitters^16,21–23^, recent studies in the Arctic have provided evidence to the contrary. For example, an increased N input has been shown to induce the emission of N_2_O from peat due to the decline of oligotrophic mosses^24^; specific areas of thermokarst (permafrost collapse due to ground ice melt) can be active N_2_O emitters, particularly in the part of thermo-erosion gullies^25^; and peat circles (bare peat surfaces) emit N_2_O at high rates during the growing season (1.2–20 mg N m^−2^ d^−1^)^19^. Thus, further research to elucidate how Arctic tundra soils contribute to N_2_O emissions to the atmosphere is needed.

Bird colonies accelerate soil N turnover via continuous inputs of nutrient-rich labile organic matter in the form of bird-derived faeces, feathers, egg shells, and carcasses. Consequently, bird-affected soils are high emitters of nitrogenous gases such as ammonia^26–29^ and N_2_O^30–33^. In the High Arctic tundra, taluses (steep slopes) at the bottom of cliffs inhabited by seabirds are known to be the area, which receives intensive organic matter inputs^34–36^. Such taluses are covered by vegetation consisting of mosses and compact vascular plants^34^. Taluses are formed from debris that has been fallen from the cliff and are therefore well drained topographically; however, because mosses are the parent material of the surface soil on bird-affected taluses, this soil has a high water retentivity^37^. Thus, bird-affected taluses can become denitrification and N_2_O emission hotspots because an anaerobic condition is developed in the moist surface soils and the continuous supply of labile organic matter and nitrate provided by mineralisation and nitrification facilitates denitrification. Furthermore, the mixture of drained and moist conditions produces a partial anaerobic environment that promotes the excess N_2_O production.

**Figure 1:**
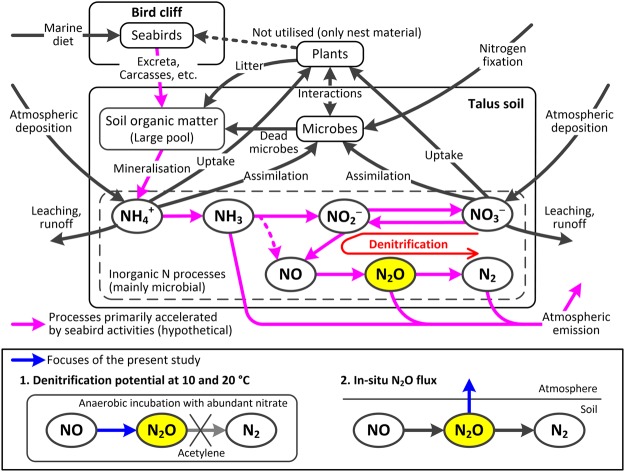
Upper panel: Proposed scheme of accelerated nitrogen turnover in seabird-affected talus soil in the High Arctic. Lower panel: Focuses of the present study.

In the present study, we hypothesised that organic matter inputs from seabird cliffs in the High Arctic accelerate N turnover in talus soil, which produces denitrification hotspots with possible N_2_O emissions (Fig. 1). Specifically, we hypothesised that (1) the surface soil on seabird-affected taluses has a high denitrification potential, (2) seabird-affected taluses emit N_2_O to the atmosphere, and (3) these denitrification potentials and N_2_O emissions decrease with increasing distance from the bottom of the cliff in association with the decrease in organic matter input.

**Figure 2:**
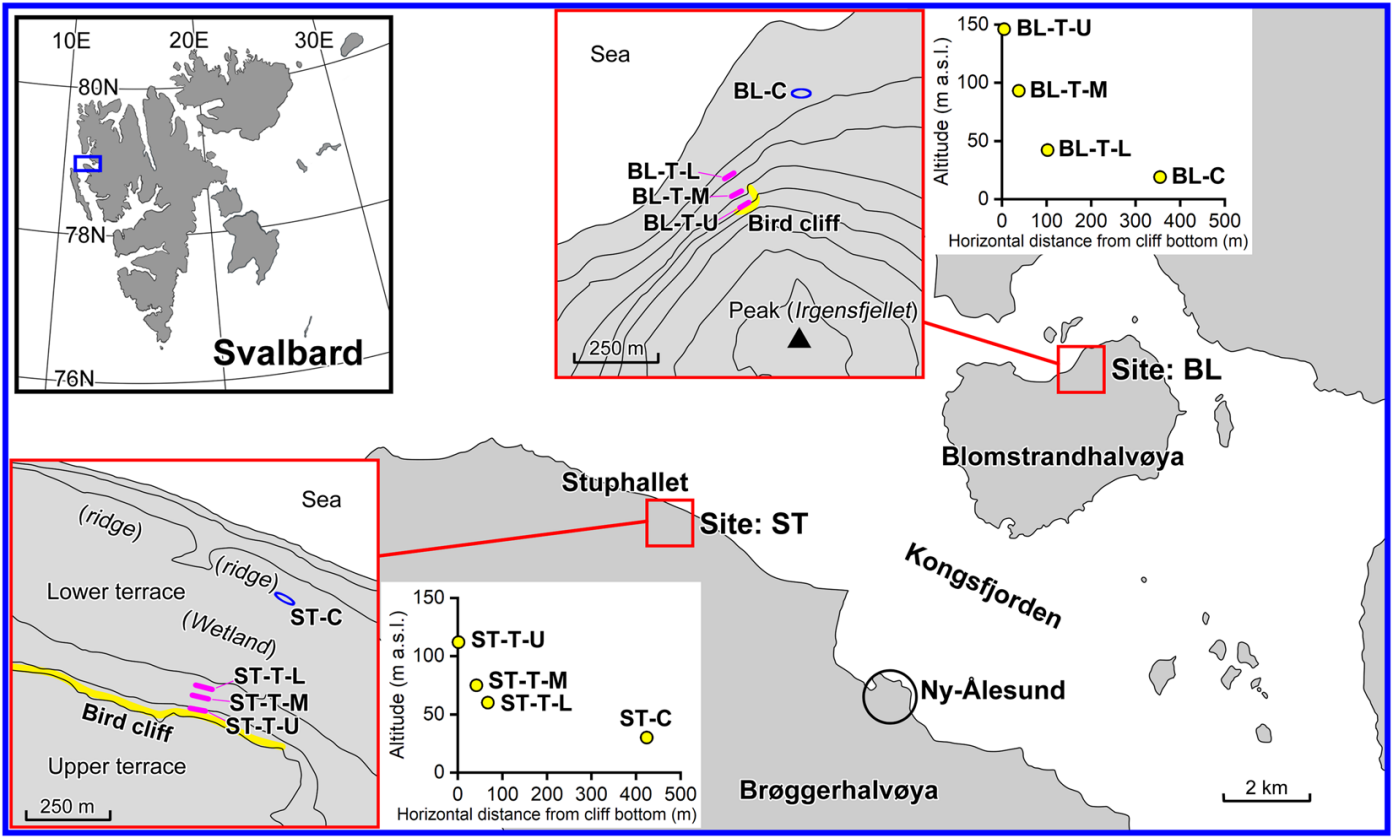
Study area and study sites. BL, study site in Blomstrandhalvøya; ST, study site in Stuphallet; T-U, talus (upper part); T-M, talus (middle part); T-L, talus (lower part); C, control area not directly affected by seabirds.

Denitrification potential corresponds to the maximum N_2_O production rate under a strong anaerobic condition in the presence of abundant substrates and at a constant temperature. To determine denitrification potentials, *in-situ* N_2_O emissions, and other relevant properties of seabird-affected talus soil, we conducted field surveys at two sites, one in Blomstrandhalvøya (BL) and the other in Stuphallet (ST), near Ny-Ålesund, Svalbard, in the European High Arctic (Fig. 2; Supplementary Information). Each site comprises a talus at the base of a cliff inhabited by breeding piscivorous seabirds and a control area not affected by seabirds. The field survey was conducted in July during the nightless midsummer when ca. 400 pairs of black-legged kittiwakes (*Rissa tridactyla*) and a few northern fulmars (*Fulmarus glacialis*) inhabited the cliff at the BL site and ca. 10 pairs of Atlantic puffins (*Fratercula arctica*) and ca. 5 pairs of northern fulmars inhabited on the cliff at the ST site.

## Results

### Denitrification potentials

**Figure 3:**
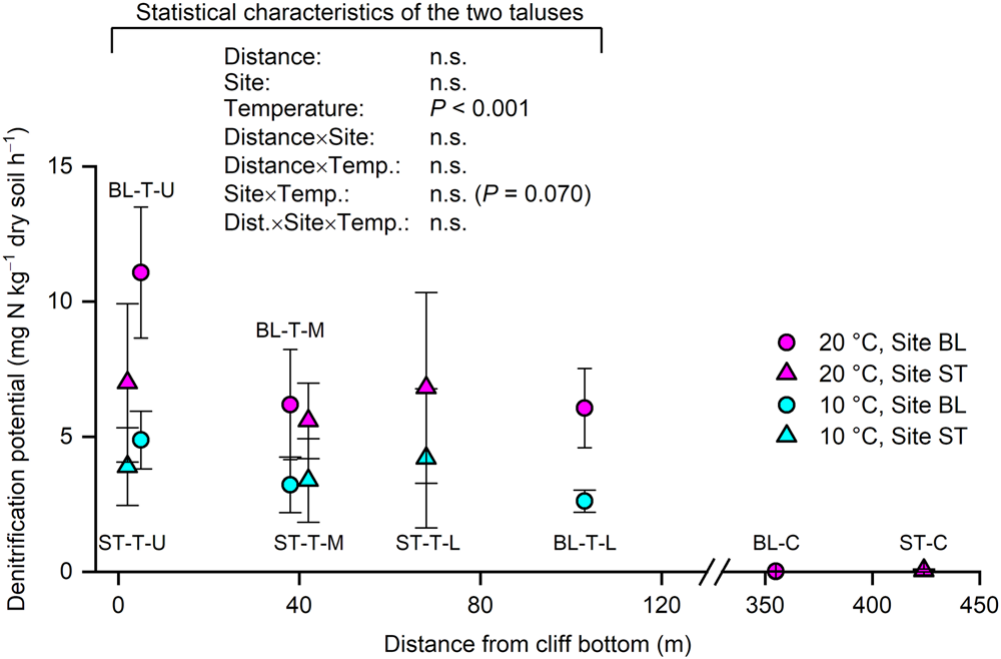
Denitrification potentials of the soils (0–5 cm depth) at 10 and 20 °C. Bars denote standard deviation (*n* = 3). A normal distribution assumption was accepted for the talus soil data. BL, study site in Blomstrandhalvøya; ST, study site in Stuphallet; T-U, talus (upper part); T-M, talus (middle part); T-L, talus (lower part); C, control area not directly affected by seabirds; n.s., not significant.

The talus soils (0–5 cm depth) at both BL and ST had high denitrification potentials at all locations (upper, middle, and lower parts of the taluses; Fig. 2; Supplementary Information) compared with those at control plots (Fig. 3). Denitrification potentials were measured at 10 and 20 °C, with the former temperature approximating the mean daily maximum air temperature in July in Ny-Ålesund and the latter being used to examine how the soils responded to higher-than-usual temperatures. The mean denitrification potentials for the soils from the three parts of the taluses at 10 °C were 2.62–4.88 and 3.38–4.20 mg N kg^−1^ dry soil h^−1^ at BL and ST, respectively, and those at 20 °C were 6.06–11.1 and 5.59–6.99 mg N kg^−1^ dry soil h^−1^ at BL and ST, respectively; significant positive temperature responses (*P* < 0.001) were observed with Q10 values of 1.9–2.3 at BL and 1.6–1.8 at ST. The mean denitrification potentials of the soils in the control areas were a few hundredths of the values recorded for the talus soils (Fig. 3). At the two taluses, the effect of distance from the cliff bottom (distance) on denitrification potential and the interaction between site and incubation temperature were not significant (Fig. 3).

### ***In-situ*** N_2_O emissions

**Figure 4:**
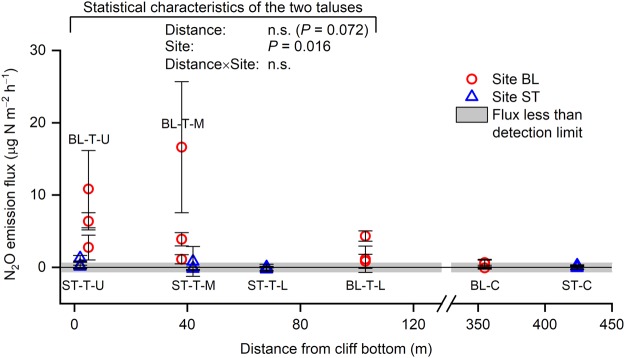
*In-situ* N_2_O emission flux. Bars denote standard deviation (*n* = 3). A log-normal distribution assumption with Yeo–Johnson transformation was accepted for the talus soil data. BL, study site in Blomstrandhalvøya; ST, study site in Stuphallet; T-U, talus (upper part); T-M, talus (middle part); T-L, talus (lower part); C, control area not directly affected by seabirds; n.s., not significant.

*In-situ* N_2_O emissions from undisturbed soil surfaces were recorded at the talus at BL, but were mostly below the detection limit at the talus at ST and the two control areas (Fig. 4). The difference in N_2_O emissions between the two taluses was significant (*P* = 0.016). The mean N_2_O emissions at the three parts of the talus at BL were in the range of 2.1–7.2 µg N m^−2^ h^−1^, with the highest emission (16.6 µg N m^−2^ h^−1^) recorded at a plot on the middle part of the talus (Fig. 4). The effect of distance on N_2_O emission was not significant (*P* = 0.072) (Fig. 4).

### Soil properties related to N turnover

**Figure 5:**
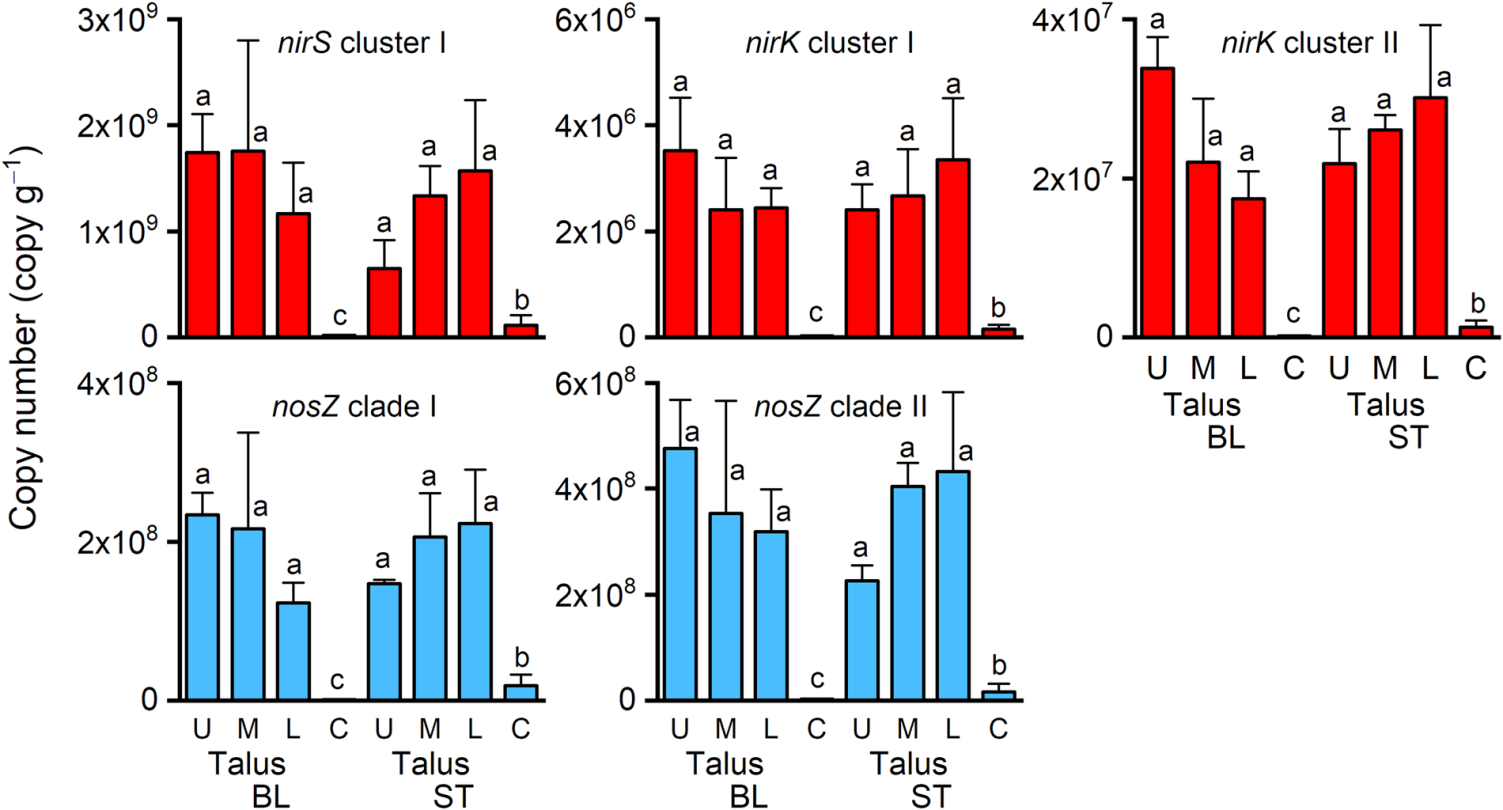
Copy numbers of denitrifier genes encoding nitrite reductase (*nirS* cluster I, *nirK* cluster I, and *nirK* cluster II) and nitrous oxide reductase (*nosZ* clade I and *nosZ* clade II) in soils. Bars denote standard deviation (*n* = 3). Different letters denote a significant difference (*P* < 0.05) under a log-normal distribution assumption. BL, study site in Blomstrandhalvøya; ST, study site in Stuphallet; U, talus (upper part); M, talus (middle part); L, talus (lower part); C, control area not directly affected by seabirds.

The talus soils at both sites were organic soils originating from plant litter, mostly mosses, with the following characteristics favourable for denitrification compared with those of the soils in control areas: a high water content (58.5–82.0% w/w), which promotes the development of anaerobic conditions, and high total C content (20.5–38.8%) and total N content (1.79–2.77%), which suggests a high input of organic matter (Table 1). In addition, the talus soils had a low bulk density (127–513 kg m^−3^), near-neutral pH (7.1–7.4), and a very low nitrite content (data not shown). The two talus soils also had a high denitrification gene content (*nirS* in cluster I, *nirK* in clusters I and II [*nir*: nitrite reductase], and *nosZ* in clades I and II [*nos*: N_2_O reductase]; Fig. 5), suggesting that active denitrification was on-going in the talus soils. In contrast, the soils at the two control areas were mineral soils with lower water, total C, total N, and inorganic N content, higher bulk density (Table 1); and less denitrification gene content (Fig. 5) compared with the talus soils.

**Table 1:** Soil properties.

Site	BL				ST			
Location	T-U	T-M	T-L	C	T-U	T-M	T-L	C
Soil water content (% w/w)	60.5^ab^ (7.8)	60.1^ab^ (17.8)	58.5^b^ (8.4)	1.7^c^ (1.2)	66.1^ab^ (9.6)	79.0^ab^ (0.6)	82.0^a^ (2.5)	20.7^c^ (0.5)
Bulk density (kg m^−3^)	509^b^ (267)	440^b^ (188)	513^b^ (109)	1840^c^ (167)	373^b^ (214)	127^a^ (21)	131^a^ (6)	1400^c^ (70)
Soil pH (H_2_O)	7.3^a^ (0.3)	7.1^a^ (0.3)	7.4^a^ (0.2)	7.3^a^ (0.5)	7.3^a^ (0.3)	7.3^a^ (0.2)	7.1^a^ (0.2)	7.0^a^ (0.2)
Total carbon (%)	25.4^ab^ (3.1)	29.6^ab^ (8.5)	20.5^b^ (7.0)	0.18^c^ (0.08)	28.6^ab^ (6.9)	38.8^a^ (3.1)	36.2^a^ (3.8)	2.6^c^ (0.8)
Total nitrogen (%)	2.57^a^ (0.40)	2.77^a^ (0.54)	1.79^a^ (0.54)	0.012^b^ (0.006)	2.36^a^ (0.64)	2.40^a^ (0.14)	2.37^a^ (0.35)	0.23^b^ (0.05)
Carbon-to-nitrogen ratio	9.9^a^ (0.6)	10.6^a^ (1.1)	11.4^ab^ (0.7)	16.3^c^ (1.5)	12.3^abc^ (2.4)	16.2^c^ (1.4)	15.4^bc^ (2.2)	11.2^a^ (0.9)
Ammonium content (mg N kg^−1^)	6.92^abc^ (1.79)	6.14^abc^ (3.13)	3.91^bc^ (0.89)	0.30^c^ (0.14)	13.5^a^ (4.75)	8.22^ab^ (4.23)	1.23^bc^ (2.12)	ND
Nitrate content (mg N kg^−1^)	2430^a^ (603)	2270^a^ (1380)	885^ab^ (397)	0.30^c^ (0.25)	165^b^ (130)	51.4^b^ (19.6)	21.2^b^ (3.6)	1.29^c^ (0.46)
δ^15^N of bulk soil (‰)	14.1^ab^ (1.6)	19.4^a^ (2.4)	21.3^a^ (1.4)	1.5^d^ (0.3)	10.7^b^ (2.2)	6.3^c^ (0.9)	5.9^c^ (1.3)	4.6^c^ (0.6)

Values in parentheses denote the standard deviation (*n* = 3). Different letters within each row (a–d) denote a significant difference among locations (*P* < 0.05) under the assumption of a log-normal distribution for bulk density, nitrate content, and δ^15^N and a normal distribution for the other soil properties. ND, not detected. BL, study site in Blomstrandhalvøya; ST, study site in Stuphallet; T-U, talus (upper part); T-M, talus (middle part); T-L, talus (lower part); C, control area not directly affected by seabirds.

However, the two talus soils were not identical. The carbon-to-nitrogen (CN) ratios of the soils on the middle and lower parts of the talus at ST (15.4–16.2) were significantly higher than those of the soils collected at BL (9.9–11.4; *P* < 0.05; Table 1). The nitrate content of the soils on the upper and middle part of the talus at BL (2270–2430 mg N kg^−1^ dry soil), which accounted for 8–9% of the total N content, were significantly higher than those at ST (21.2–165 mg N kg^−1^ dry soil; *P* < 0.05; Table 1). The δ^15^N values (stable N isotope ratio [^15^N/^14^N] expressed in δ notation) of the soils on the middle and lower parts of the talus at BL (19.4–21.3‰) were significantly higher than those at ST (5.9–10.7‰; *P* < 0.05; Table 1).

**Figure 6:**
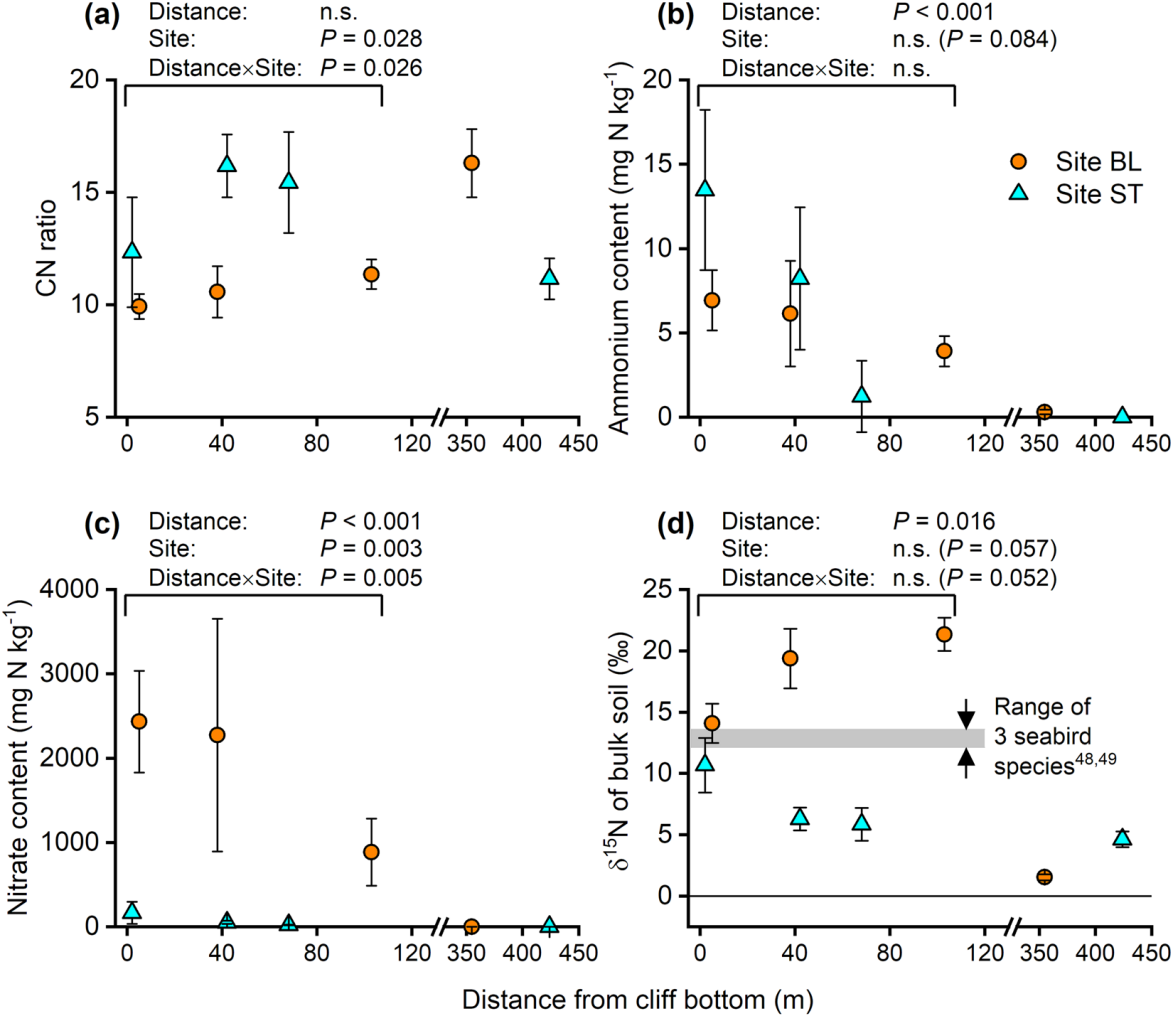
(**a**) Carbon-to-nitrogen (CN) ratio, (**b**) ammonium content, (**c**) nitrate content, and (**d**) δ^15^N of soils (0–5 cm depth). Bars denote standard deviation (*n* = 3). A normal distribution assumption was accepted for CN ratio and ammonium content, and a log-normal distribution assumption was accepted for nitrate content and δ^15^N. BL, study site in Blomstrandhalvøya; ST, study site in Stuphallet; n.s., not significant. The grey bar shows the δ^15^N range for lipid or liver tissue of three seabird species (black-legged kittiwake, northern fulmar, and Atlantic puffin).

At the two taluses, the effect of distance was not significant for CN ratio but was significant for ammonium content (*P* < 0.001), nitrate content (*P* < 0.001), and δ^15^N (*P* = 0.016) (Fig. 6). The effect of site was significant for CN ratio (*P* = 0.028) and nitrate content (*P* = 0.003) and not significant for ammonium content (*P* = 0.084) and δ^15^N (*P* = 0.057) (Fig. 6). The interaction between distance and site was significant for CN ratio (*P* = 0.026), which was attributed to the sharper increase in CN ratio with increasing distance from the cliff bottom at ST than at BL, and nitrate content (*P* = 0.005), which was attributed to the sharper decrease in nitrate content with increasing distance from the cliff bottom at BL than at ST, not significant for δ^15^N (*P* = 0.052) and ammonium content (Fig. 6).

## Discussion

### High denitrification potentials of talus soils

The talus soils had high dentification potentials at both sites (Fig. 3); this result supported our first hypothesis. However, the effect of distance from the cliff bottom on denitrification potential was not significant (Fig. 3); this part of results was contrary to third hypothesis. The denitrification potentials of the talus soils at BL and ST (2.62–4.88 [10 °C] and 5.59–11.1 [20 °C] mg N kg^−1^ dry soil h^−1^) were very high compared with the following literature values: 0.1 (meadow snowbed) to 1.6 (mesic heath) mg N kg^−1^ dry soil h^−1^ at room temperature for tundra soils collected in northern Sweden^9^, 0.78 (cryoturbated soil) and 0.50 (unturbated soil) mg N kg^−1^ dry soil h^−1^ at 20 °C collected in a Russian discontinuous permafrost zone^38^, 0.51 (surface fen soil) mg N kg^−1^ dry soil h^−1^ at 20 °C collected in northeast Finland^39^, and 0.0056–1.74 mg N kg^−1^ h^−1^ at 25 °C for 26 topsoil (0–20 cm) samples collected at farmlands in Shanxi province and Inner Mongolia, China^40^. Thus, the high denitrification potentials (Fig. 3) and abundance of denitrification genes (Fig. 5) in the present talus soils indicate that the bird-affected taluses at the study sites are denitrification hotspots within the High Arctic. We did not observe a significant change in denitrification potential with increasing distance from the cliff bottom (Fig. 3); we attributed this finding to the high water and total C contents (Table 1), which are properties advantageous to denitrification, of the soils throughout the length of the taluses. Note that the denitrification potentials were determined in the presence of abundant nitrate and that real-world denitrification activities depend on local nitrate availability.

The positive temperature responses of the denitrification potentials of the talus soils (Fig. 3) denote that warming might accelerate *in-situ* denitrification of the talus soils. The Q10 values of the talus soils, 1.9–2.3 at BL and 1.6–1.8 at ST, are comparable with that reported from Swedish lake sediment (1.69)^41^. Of the two temperatures examined, the lower temperature (10 °C) was close to the mean maximum daily air temperature in Ny-Ålesund in July (warmest month in the year) for the decade from 2008 to 2017 (8.1 °C)^42^; however, the higher temperature (20 °C) was higher than the maximum air temperature recorded in the same period (14.8 °C)^42^. Thus, the positive temperature response indicates that future climate warming might promote denitrification in bird-affected talus soils in the High Arctic.

### Marked N_2_O emissions only at BL

The talus at BL emitted N_2_O and the talus at ST was a poor N_2_O emitter (Fig. 4). These results partly supported our second hypothesis. The effect of distance from the cliff bottom on N_2_O emission was not significant contrary to third hypothesis (Fig. 4). The *in-situ* N_2_O emissions recorded at the talus at BL (0.85–16.6 µg N m^−2^ h^−1^; mean ± SD, 5.3 ± 5.3 µg N m^−2^ h^−1^; *n* = 9 plots) challenge the conventional understanding that Arctic soils are poor N_2_O emitters^16,21–23^. The N_2_O emissions from the talus at BL were smaller than the highest reported N_2_O emitters in the Arctic: 50–820 µg N m^−2^ h^−1^ from peat circles in subarctic eastern European tundra throughout the growing season from June to October^19^ and 20.9 ± 3.9 µg N m^−2^ h^−1^ from a bird-affected, flat, semi-wet tundra with high bird activity (a bird sanctuary inhabited mainly by Arctic terns [*Sterna paradisaea*] and barnacle geese [*Branta leucopsis*]) in Ny-Ålesund from late July to early August^30^. However, they are comparable with those reported for medium- and low-bird activity areas (4.8 ± 2.5 and 4.5 ± 2.4 µg N m^−2^ h^−1^, respectively) in the flat tundra in Ny-Ålesund^30^.

In tundra soil, the main pathway of N_2_O production is denitrification under intermediate or high moist soil conditions^18,19,38,39^, although sufficient nitrate must also be present^18,19^. Furthermore, for N_2_O to be emitted, there must be surplus of N_2_O produced. Soil moisture has a major effect on the availability of oxygen to soil microbes, and there is an optimum wetness to facilitate surplus N_2_O production^11^, which is intermediately moist conditions to promote both aerobic nitrification and anaerobic denitrification^19,25^. Excessively moist conditions promote complete denitrification where N_2_O is reduced to N_2_ and therefore there is no surplus N_2_O to emit^11^. There is a contradictory finding that nitrification is the main pathway of N_2_O production in the Canadian Arctic tundra^43^, which is a result of competition between denitrifiers and fungi for nitrate that increases the relative contribution of nitrification to N_2_O emissions^44^. Since the talus soils at BL contained high amounts of nitrate (Table 1), we conclude that denitrification is the main pathway of N_2_O production in the talus soils owing to the abundance of substrate; however, nitrification supplying the abundant nitrate might also contribute to N_2_O production. Further research is needed to elucidate contributions of nitrification and denitrification to N_2_O emitted from bird-affected taluses to the atmosphere.

**Figure 7:**
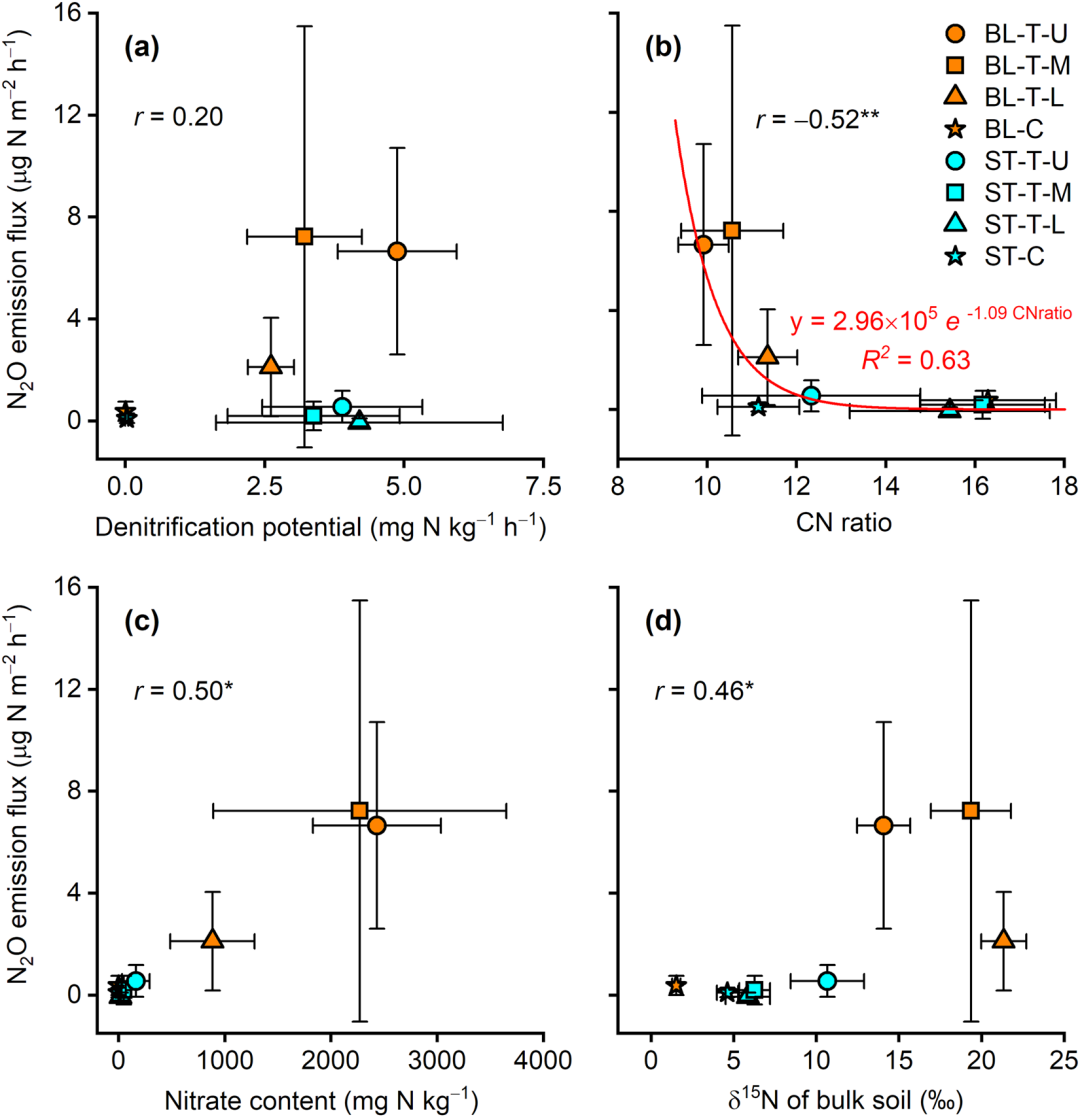
Relationship between *in-situ* N_2_O emission flux and (**a**) denitrification potential, (**b**) carbon-to-nitrogen (CN) ratio, (**c**) nitrate content, and (**d**) δ^15^N of bulk soil. Bars denote standard deviation (*n* = 3). *P < 0.05; **P < 0.01. BL, study site in Blomstrandhalvøya; ST, study site in Stuphallet; T-U, talus (upper part); T-M, talus (middle part); T-L, talus (lower part); C, control area not directly affected by seabirds.

The marked *in-situ* N_2_O emissions observed at the talus at BL (Fig. 4) indicate the presence of a mixture of aerobic and anaerobic conditions. This interpretation is supported by the active denitrification, active nitrification, and rapid N turnover in the talus soils at BL. Active denitrification in the talus soils at BL was confirmed by the high denitrification potentials (Fig. 3) and the abundance of denitrification genes (Fig. 5) in the soils; however, the talus soils at ST were also characterised by active denitrification. Denitrification potential does not predict *in-situ* N_2_O emission, as shown by the lack of correlation between the two variables (Fig. 7a). Active nitrification in the talus soils at BL was confirmed by the high nitrate content in the soils (Table 1), particularly at the upper and middle parts of the talus (2270–2430 mg N kg^−1^ dry soil). These values are higher than reported values (ca. 480 mg N kg^−1^) for soils in southwest Svalbard beneath a colony of ca. 10,000 breeding pairs of Brünnich’s guillemots (*Uria lomvia*) and black-legged kittiwakes^36^, and is comparable with values (ca. 1452 mg N kg^−1^) for soils collected from a breeding colony of black-tailed gulls (*Larus crassirostris*) in Japan with high N_2_O emissions^45^ and from a year-round colony of great cormorants (*Phalacrocorax carbo*) in Japan (ca. 3530 mg N kg^−1^) with extremely high N_2_O emissions^33^. The correlation between *in-situ* N_2_O emission and nitrate content was significant and positive (*P* = 0.013; Fig. 7c). Rapid N turnover in the talus soils at BL was confirmed by the low CN ratios and high δ^15^N values for the soils (Table 1). High nutrient input accelerates organic matter turnover in tundra soil, which decreases the soil CN ratio^6^. The CN ratios of the talus soils at BL (9.9–11.4) were lower than those at ST (12.3–16.2), which indicates that there is faster organic matter turnover at BL than at ST (Table 1). N_2_O emissions are negatively correlated with soil CN ratio in European forest soils^11^. Similarly, the present data showed a significant negative correlation between *in-situ* N_2_O emission and CN ratio (*P* = 0.009; Fig. 7b). In German and Finnish histosols, annual N_2_O emissions are negligible at a CN ratio higher than 25, but below this threshold N_2_O emissions increase exponentially with decreasing CN ratio^18^. Such a negative exponential relationship was also found in the present data (*R*^2^ = 0.63; Fig. 7b), where N_2_O emissions were below the detection limit (0.6 µg N m^−2^ h^−1^; Fig. 4) at CN ratios greater than 12. The δ^15^N value of bulk soil increases with inorganic N loss from the soil via nitrate leaching and atmospheric emissions of ammonia, N_2_, and N_2_O^46^. The δ^15^N values of soils from the middle and lower parts of the talus at BL were higher than those of the seabird species that inhabited the cliffs (Fig. 6d). Such enrichment of ^15^N indicates active inorganic N loss, that is, there is rapid inorganic N turnover in the talus soils at BL. The present data showed a significant positive correlation between *in-situ* N_2_O emission and the δ^15^N value of bulk soil (*P* = 0.024; Fig. 7d).

We conducted our sampling in July because we wanted to examine the conditions at the time during the year when N_2_O emissions are greatest, and the maximum N_2_O emissions from peat circles^19^ and bird-affected flat tundra^30^ were recorded in July. The air temperature during our field surveys were 8–9 °C at BL and 7–8 °C at ST, and these temperatures were comparable with the mean daily maximum air temperature in July in Ny-Ålesund (8.1 °C)^42^. The present data were obtained close to the average daily maximum air temperature. However, our snapshot measurements in the present study do not support the conclusion that the talus at BL always emits N_2_O and the talus at ST never emits N_2_O.

### Effects of seabirds on soil N turnover

The differences in soil properties between the two taluses, particularly with respect to the lower CN ratio, much higher nitrate content, and higher δ^15^N at BL (Table 1), are attributable to the approximately 30-fold larger seabird density at BL compared with that at ST. It is natural that there is a quantitative difference in organic matter input due to the difference in bird density.

Piscivorous and planktivorous seabirds promote the growth of different plant species near their colonies and the total N content and δ^15^N values of plants are higher near piscivorous colonies than near planktivorous colonies^47^. This implies that piscivorous seabirds accelerate N turnover of the local soil more than planktivorous seabirds do. The three seabird species inhabiting our study sites are all piscivorous; however, these birds also feed on zooplankton, such as amphipods and krill, depending on prey availability^48,49^. The δ^15^N of liver tissue of black-legged kittiwake, northern fulmar, and Atlantic puffin in Iceland has been reported as 13.2–14.2‰, 13.5–14.0‰, and 12.2–13.0‰, respectively^48^, and that of lipids of black-legged kittiwake and northern fulmar in northwest Svalbard as 12.1‰ and 13.6‰, respectively^49^. In contrast, the faeces of little auk (*Alle alle*), a planktivorous seabird, in southwest Svalbard has a lower δ^15^N of 8.0‰^34^. Seabirds might alter their diet to adapt to environmental changes. In northwest Svalbard, the diet of black-legged kittiwakes is shifting from Arctic to Atlantic species due to recent climate warming^50^. Population and colonisation of High Arctic seabirds might be also changing due to climate warming and environmental changes. Seabirds have an important role as a nutrient carrier from marine to terrestrial ecosystems; therefore, further research is needed to elucidate how changes in seabird activities impact seabird-affected talus soils as denitrification hotspots and possible N_2_O emitters in the High Arctic.

## Methods

### Study sites

We selected two study sites near Ny-Ålesund, Svalbard, in the European High Arctic (Fig. 2). Detailed information of the two study sites is shown in Supplementary Information.

### Field survey

Three lines on the upper, middle, and lower parts of each talus were set in relation to the distance from the cliff bottom. A control area unaffected by the seabirds was also set at each site for comparison with the characteristics of the talus soils. At each location (three lines on each talus and control areas), three plots were set 10 to 15 m apart. Gas and soil samples were collected from three points within each plot (Supplementary Information).

The field survey was conducted on 21 July 2015 at the talus and control area at BL, on 12 July 2017 at the upper and middle parts of the talus at ST, and on 15 July 2017 at the lower part of the talus and control area at ST. Gas and soil samples were collected from the same points to enable direct comparison between *in-situ* N_2_O emissions and soil-related variables including denitrification potential. We used a closed-chamber technique^51^, downsized and simplified, to first collect gas samples and then to collect soil samples. The chamber was made by connecting two stainless-steel soil cores (volume, 100 mL each; DIK-1801, Daiki, Saitama, Japan) using polyvinylchloride adhesive tape, and the upper and lower cores were used for gas and soil sampling, respectively. This simple method was effective for conducting multipoint sampling on the steep, uneven surfaces of the taluses, and the small chambers reduced environmental disturbance due to the destructive nature of the soil sampling. The sampling procedure is explained in detail in the Supplementary Information.

### Measurement of denitrification potential

The denitrification potential of the soil samples was determined by using an acetylene inhibition technique^9,38–41,52–54^. Briefly, 8 g of fresh soil and 12 mL of substrate solution were added to a glass vial (volume, 140 mL). The substrate solution contained 1 mM nitrate as the denitrification substrate and 0.1 mM chloramphenicol as an inhibitor of cell growth to eliminate apparent increases in the N_2_O production rate due to cell growth^52^. The vial was purged with N_2_ to produce a strong anaerobic condition and then 10% of the vial air volume was substituted with purified acetylene to inhibit the final stage of denitrification from N_2_O to N_2_. No additional organic matter as an electron donor for denitrification was added because the talus soils were rich in organic matter. The vials were incubated at 10 or 20 °C for 6 h, and gas sampling was conducted at 2, 4, and 6 h from the start of incubation. The N_2_O mixing ratio in the gas samples was determined by using a gas chromatograph equipped with an electron capture detector (GC-14A, Shimadzu, Kyoto, Japan). N_2_O production rates between 2–4 h and 4–6 h were averaged to obtain the denitrification potential for the sample.

Our use of the acetylene inhibition technique allows comparisons to be made between our results and those of other studies that use the same technique^9,38–40^. In this technique, N_2_O is originated only by denitrification because the anaerobic conditions and acetylene inhibit nitrification^53^. The acetylene inhibition technique is a common means of determining denitrification potential^41,52,55^; however, possible underestimation has been reported due to incomplete inhibition of N_2_O reduction^40,54^ arising from incomplete diffusion of the acetylene into soil microsites, microbial consumption of the acetylene, and differences in acetylene sensitivity among the denitrifiers in the soil^40^. We addressed the issue of incomplete diffusion of acetylene into soil microsites by using vigorous shaking during incubation, and we consider microbial consumption of acetylene to have been negligible during the short-term (6 h) anaerobic incubation^40^. Although the current knowledge regarding the relationship between denitrifying flora and denitrification potential of Arctic soil is incomplete, experiments showed that the bias of denitrification potentials determined by the acetylene inhibition technique is lower in high-fertility soils^40^. The talus soils in the present study were rich in total C and total N (Table 1) and were therefore of high fertility, suggesting that the determined denitrification potentials had low biases; we therefore concluded that the present data sufficiently approximated the true denitrification potential.

### Determination of ***in-situ*** N_2_O emissions

The N_2_O mixing ratio in the collected gas samples was determined by gas chromatography, as described in the previous section. *In-situ* N_2_O emissions were calculated by using the rate of change in N_2_O mixing ratio inside the chamber, atmospheric pressure, air temperature, chamber volume, and soil surface area^51^. The detection limit of N_2_O flux was 0.0 ± 0.6 μg m^−2^ h^−1^, which corresponded to the 10-fold standard deviation (two-sided) of the mixing ratio for a standard N_2_O gas (300 ppb).

### Soil analyses

Fresh soil samples were used to determine soil water content (w/w), bulk density, and soil pH (H_2_O). Soil water content was determined using a dry oven. Bulk density was calculated from the initial soil weight and the soil water content. Soil pH was measured by using a pH meter (B-712, Horiba, Tokyo, Japan). Air-dried soil samples were used to determine total C, total N, ammonium, nitrite, and nitrate content and δ^15^N. Total C and total N content were measured by using an NC analyser (Sumigraph NC-22F, Sumika Chemical Analysis Service, Tokyo, Japan). Inorganic N content was determined by using a flow injection analyser (AQLA-1000, Aqualab, Tokyo, Japan) for which 5 g of fresh soil was extracted with 40 mL of 10% KCl solution. δ^15^N was determined with an elemental analyser/isotope ratio mass spectrometer (Flash EA 1112 coupled to a Delta V Advantage via ConFlo III interface, Thermo Fisher Scientific, Bremen, Germany). Soil samples containing ca. 45 µg N were placed in tin capsules, loaded into a Costech Zero-Blank Autosampler (Costech, Valencia, CA, USA), and then introduced to the reactor furnace of the elemental analyser. δ^15^N was determined as (^15^N/^14^N)_sample_/(^15^N/^14^N)_standard_−1, where ‘standard’ refers to international standards of known ^15^N/^14^N ratios calibrated against N_2_ in air. The analytical reproducibility of this method was 0.2‰.

To examine the abundance of denitrification genes in soil samples, DNA was extracted from 0.4 g of soil sample by using a FastDNA SPIN Kit for Soil (MP Biomedical, Santa Ana, CA, USA) in accordance with the methods of Hayashi *et al*.^56^. Then, the polymerase chain reaction (PCR) was used to determine the existence of gene sequences encoding target denitrification enzymes in the DNA extracted from the soil samples by using primers targeting the nitrite reductase-encoding genes *nirK* (clusters I, II, III, and IV) and *nirS* (clusters I, II, and III) and the nitrous oxide reductase-encoding gene *nosZ* (clades I and II)^57^ in accordance with the methods of Wang *et al*.^58^. The detected genes were subjected to quantitative PCR analysis using SYBR Premix Ex Taq (Takara Bio, Kyoto, Japan) and a StepOnePlus Real-Time PCR system (Thermo Fisher Scientific) in accordance with the methods of Wang *et al*.^58^. A 1-μL sample of 10-fold diluted soil DNA was used as the template in a reaction volume of 20 μL.

### Statistical analysis

Tests of statistical significance for the denitrification potential of the talus soils were performed by using a mixed model (‘proc mixed’ ver. 9.4) in the SAS software (SAS Institute Inc., Cary, NC, USA) to assess the effects of distance, site, incubation temperature, and their interactions, for which α = 0.05 was used as the limit of significance. Distance was treated as a covariate. Location and sampling plot, nested within each location (Supplementary Information), were treated as random effects. Variance components were estimated by using the restricted maximum likelihood method. Effects of distance and site, and their interaction, on *in-situ* N_2_O emission from talus were tested using the SAS mixed model but with the additional random effect of sampling point (replication) nested within each sampling plot (Supplementary Information), for which Yeo–Johnson transformation of the data using the ‘powerTransform’ function in the ‘car’ package of R^59^ was performed because some negative values showed a highly skewed distribution and violated the homoscedasticity assumption. The effects of distance and site, and their interactions, on CN ratio, ammonium and nitrate content, and δ^15^N of talus soil were similarly analysed using the SAS mixed model. For all soil properties and the denitrification gene copy number of talus and control soils, one-way ANOVA was conducted to test the effect of location by using ‘proc glm’ in SAS. Tukey’s multiple comparison was then conducted (α = 0.05) to identify significant differences among locations; logarithmic transformation was applied to the data of bulk density, nitrate content, and δ^15^N because the data violated homoscedasticity assumption. Pearson’s correlation coefficients were evaluated for the relationship between *in-situ* N_2_O emission and the variables denitrification potential, CN ratio, nitrate content, and δ^15^N. For CN ratio, a decaying exponential curve was also fitted.

## Electronic supplementary material

Supplementary Information

## Data Availability

The datasets analysed during the current study are available from the corresponding author on reasonable request.
