# Supplementary material for: Seabird-affected taluses are denitrification hotspots and potential N2O emitters in the High Arctic
Source: Sci Rep. 2018 Nov 22;8:17261. doi: 10.1038/s41598-018-35669-w (PMC6250683; doi:10.1038/s41598-018-35669-w)
Supplement: Supplementary file 1 — Supplementary Information [file 41598_2018_35669_MOESM1_ESM.pdf]

Supplementary information for

Seabird-affected taluses are denitrification hotspots and potential N_2O emitters in the High Arctic

Authors:
Kentaro Hayashi, Yukiko Tanabe, Keisuke Ono, Maarten J.J.E. Loonen, Maki Asano, Hirotsugu Fujitani, Takeshi Tokida, Masaki Uchida, Masahito Hayatsu

Study sites: Two study sites near Ny-Ålesund, Svalbard, in the European High Arctic were examined in the present study. At the site in Blomstrandhalvøya (BL), we selected a ca. 40-m-wide mountainside cliff that is shaped like a three-fold mirror and has at its base a steep talus (inclination, 36°) (Fig. S1). The talus is covered with a moss layer and compact vascular plants. Under the moss layer is an organic soil consisting mainly of moss litter and peat, and the soil on the upper part of the talus is rich in gravel and humus. Circa 400 pairs of black-legged kittiwakes (*Rissa tridactyla*) and a few northern fulmars (*Fulmarus glacialis*) inhabited the cliff during our survey in July 2015. A control area was established on bare ground ca. 300 m northeast from the bottom of the talus, which was an area covered by a glacier until the mid-20th century[1] (Fig. S1). The surface soil in the control area at BL is sandy Regosol with abundant gravel. The characteristics of the site are shown in Table S1.

At the site in Stuphallet (ST), we selected a ca. 50-m-wide section of a long talus under a ca. 2-km-long terrace cliff. The talus is steep (inclination, 32°) (Fig. S1). The talus is covered with a moss layer and compact vascular plants. Under the moss layer is organic soil consisting mainly of moss litter and peat and is occasionally rich in gravel at the upper part. There are gullies at intervals of 30 to 50 m along the long terrace cliff. Water flowing from the upper terrace has formed moss wetlands and streams on the lower terrace[2]. Water was found seeping through the talus at several places at the middle and lower parts. Circa 10 pairs of Atlantic puffins (*Fratercula arctica*) and ca. 5 pairs of northern fulmars were seen on the cliff within the study section during our field survey in July 2017. A control area was established on the hilly coastal edge of the lower terrace, which is an area covered by well-grown patchy cushion plants, mainly mountain avens (*Dryas octopetala*) and moss campion (*Silene acaulis*) (Fig. S1). The surface soil in the control area at ST is Regosol with humus. The characteristics of the site are shown in Table S1.

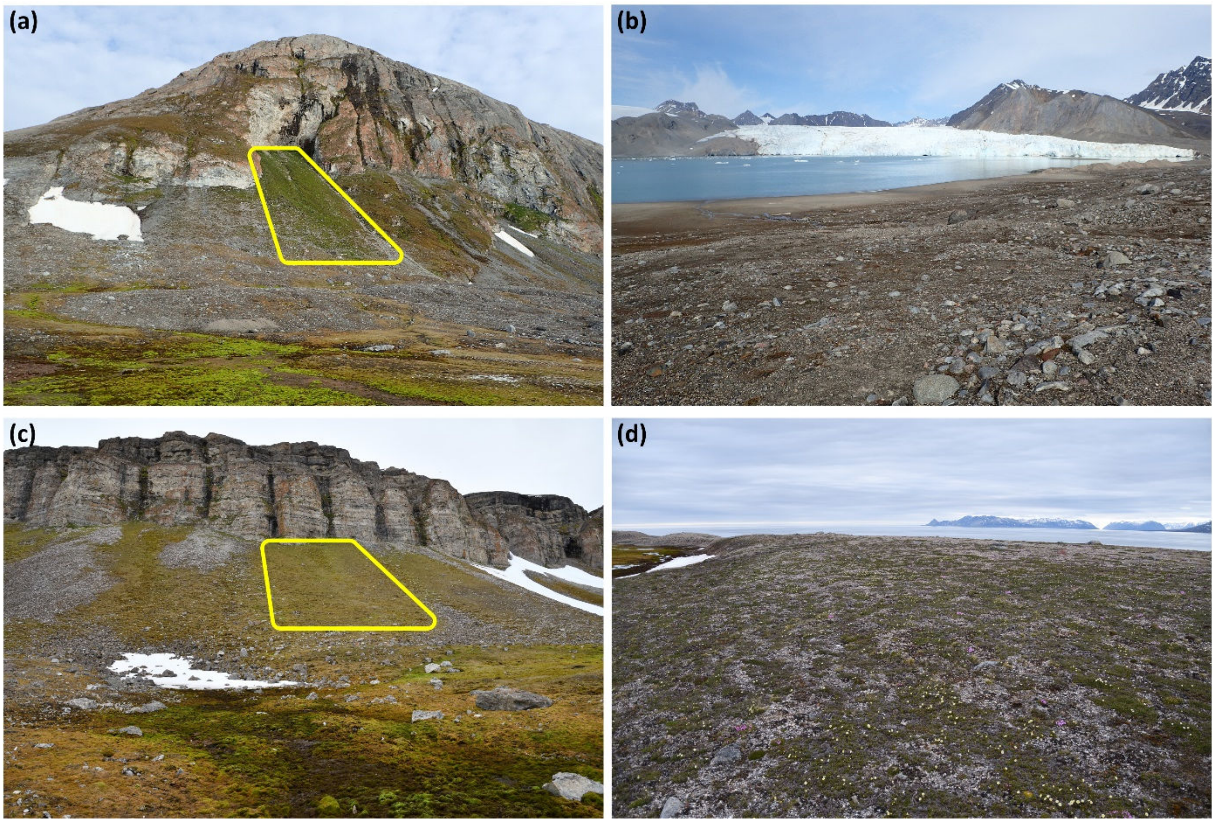

Fig. S1. Photographs of the study sites. (a) Bird-affected talus and (b) control area at Blomstrandhalvøya. (c) Bird-affected talus and (d) control area at Stuphallet.

Table S1 Characteristics of the study site

Location	Latitude	Longitude	Elevation	Vegetation	Soil	Remarks
BL-T-U	78°59′39″N	12°07′03″E	146 m a.s.l.	Moss with grass	Organic soil	Rich in gravel
BL-T-M	78°59′40″N	12°06′59″E	93 m a.s.l.	Moss with grass	Organic soil	Contains gravel
BL-T-L	78°59′42″N	12°06′55″E	42 m a.s.l.	Moss with grass	Organic soil	
BL-C	78°59′50″N	12°07′25″E	19 m a.s.l.	Bare soil	Mineral soil	Sandy
ST-T-U	78°57′27″N	11°40′02″E	112 m a.s.l.	Moss with grass	Organic soil	Rich in gravel
ST-T-M	78°57′28″N	11°40′03″E	75 m a.s.l.	Moss with grass	Organic soil	
ST-T-L	78°57′29″N	11°40′04″E	60 m a.s.l.	Moss with grass	Organic soil	
ST-C	78°57′38″N	11°40′43″E	30 m a.s.l.	Mountain avens	Mineral soil	

BL, study site in Blomstrandhalvøya; ST, study site in Stuphallet; T-U, talus (upper part); T-M, talus (middle part); T-L, talus (lower part); C, control area not directly affected by seabirds; a.s.l., above sea level.
Latitude, longitude, and elevation correspond to the values at the centre plot among the three plots in each part of the talus or control area (see Fig. S3).

References for supplementary information

1. Dallmann, W. K. Geoscience Atlas of Svalbard. [ed. Dallmann, W. K.] 292p (Norwegian Polar Institute, Report Series 148, 2015).
2. Nakatsubo, T. *et al.* Carbon accumulation rate of peatland in the High Arctic, Svalbard: Implications for carbon sequestration. *Polar Sci.* 9, 267–275 (2015).

Gas and soil sampling: A small chamber made by connecting two stainless-steel soil cores (volume, 100 mL each; height, 5 cm each) using polyvinylchloride adhesive tape (Fig. S2) was placed on the soil surface at each sampling point (Fig. S3) and the lower core of the chamber was inserted into soil to a depth 5 cm. Gas sampling was conducted 5 min after inserting the core into the soil. Gas samples were collected twice: immediately after closing the chamber with a stainless-steel cap equipped with a gas sampling tube, and again at 40 to 50 min after the chamber was closed. Polyvinylchloride adhesive tape was used to ensure airtight connections between the chamber wall and the cap. During gas sampling, atmospheric pressure, air temperature, and soil temperature were recorded. After the gas sampling, the soil inside the lower core of the sampler was collected. The soil samples collected at the taluses included fresh moss litter. Gas samples were stored in glass vials (vacuumed in advance) and transported to Japan for analysis. Soil samples collected from three points per plot were merged into a composite sample and then, with the necessary permission for export and import, transported under cold storage to Japan. A portion of the fresh soil samples was then deep-frozen at –80 °C until analysis to examine the presence of denitrification genes. Another portion of the fresh soil samples was used to determine denitrification potential, soil water content, and soil pH. The remaining soil samples were air-dried and used to determine other soil properties.

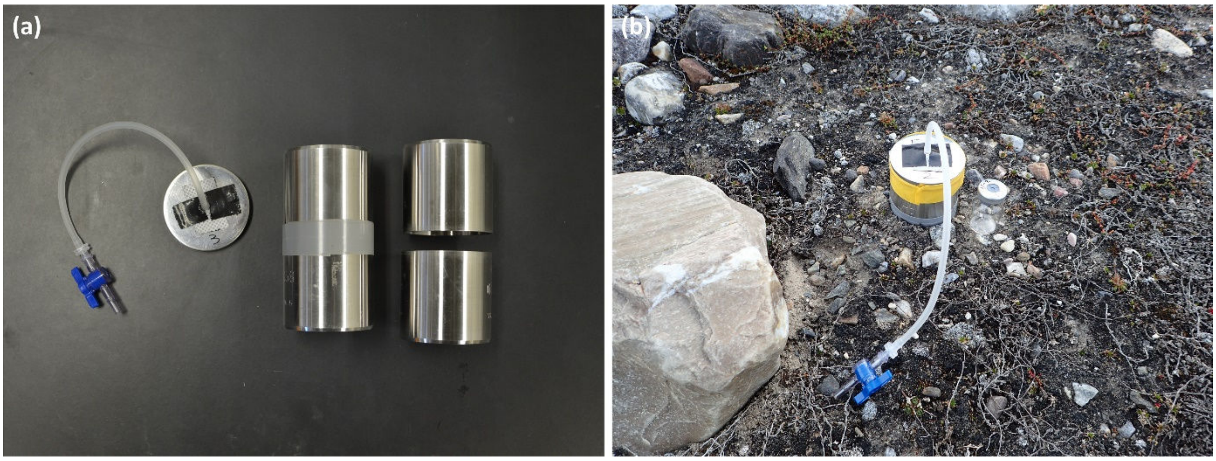

Fig. S2. Chamber design. (a) Configuration of the chmaber and (b) Installation at the field sampling.

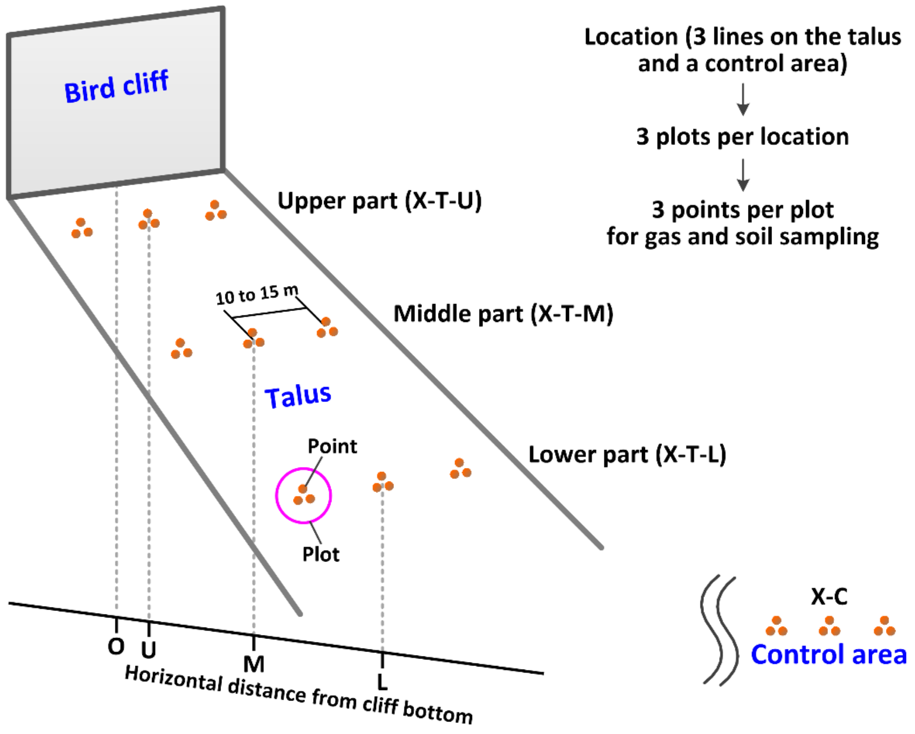

Fig. S3. Field sampling design. X denotes the site, BL (Blomstrandhalvøya) or ST (Stuphallet). T-U, location on upper part of the talus; T-M, location on the middle part of the talus; T-L, location on the lower part of the talus; C, location in a control area not directly affected by seabirds; O, cliff bottom.
